# Interactions between COVID-19 infection and diabetes

**DOI:** 10.3389/fendo.2023.1306290

**Published:** 2024-01-10

**Authors:** Hassan M. Heshmati

**Affiliations:** Endocrinology Metabolism Consulting, LLC, Hassan Heshmati and Valerie Shaw Endocrine Research, Anthem, AZ, United States

**Keywords:** coronavirus, angiotensin-converting enzyme 2, COVID-19 infection, pandemic, diabetes, immune system

## Abstract

Coronavirus disease 2019 (COVID-19) caused a major pandemic affecting human health and economy around the world since the beginning of 2020. The virus responsible for the disease is “severe acute respiratory syndrome coronavirus 2” (SARS-CoV-2). It invades the target cells by binding to angiotensin-converting enzyme 2 (ACE2). ACE2 is expressed in several organs including endocrine glands. Multiple endocrine and metabolic systems including the endocrine pancreas have been impacted by COVID-19 infection/pandemic. COVID-19 pandemic can promote obesity through alterations in lifestyle (e.g., unhealthy diet and reduced physical activity due to confinement and isolation) leading to type 2 diabetes and/or can directly impair the function of the endocrine pancreas particularly through a cytokine storm, promoting or aggravating type 1 or type 2 diabetes. The increased ACE2 receptors of high adiposity commonly associated with type 2 diabetes and the chronic hyperglycemia of diabetes with its negative impact on the immune system can increase the risk of COVID-19 infection and its morbidity/mortality. In conclusion, there are bidirectional interactions between COVID-19 pandemic and diabetes (e.g., COVID-19 infection can impact diabetes and diabetes can impact COVID-19 infection). The services offered by healthcare systems for the management of diabetes have been adapted accordingly.

## Introduction

Since the beginning of 2020, COVID-19 infection became a global crisis of the 21^st^ century, causing a major pandemic affecting human health and activities around the world and leading to a major international emergency (1–4). Several endocrine and metabolic systems including the endocrine pancreas have been impacted by this pandemic (5–9). COVID-19 infection caused a major disruption in the management of subjects with endocrine and metabolic disorders, especially those with diabetes, and the services offered by healthcare systems had to adapt accordingly and rapidly.

The purpose of this mini review is to present the interactions between COVID-19 infection and diabetes.

## Pandemic

A pandemic is an epidemic that spreads globally, crosses international boundaries, and affects large number of people. Numerous pandemics have occurred throughout the history of mankind (10, 11). The deadliest pandemics were the Plague of Justinian, the Black Death, and the Spanish Flu.

The most recent pandemic was the COVID-19 pandemic (1–3). In January 2020, Chinese authorities announced the isolation of a new type of coronavirus, SARS-CoV-2, following the occurrence in December 2019 of several pneumonia cases of unknown etiology. On March 11, 2020, the World Health Organization declared COVID-19 a pandemic.

Pandemics can influence life at individual, familial, societal, and environmental levels (12). At the individual level, there are health consequences including infection caused by the pathogen, metabolic diseases, mental disorders, impact on pre-existing conditions, and eventually death, financial consequences mainly due to unemployment, and educational consequences caused by remote learning. At the familial level, there is a risk of domestic violence due to prolonged presence of parents and children at home. At the societal level, there are major economic consequences affecting several businesses (e.g., agriculture, restaurant, hotel, store, airline, cruise, convention, concert, sport event, museum, movie, and theater) caused by limitation of social life and activities. At the environmental level, confinement may have some health benefits, at least for short term, due to a reduction in air pollution mainly secondary to decrease in circulating cars and flying planes which can also positively impact life of animals and plants.

## COVID-19 infection

### Structure and mode of action of COVID-19 virus

**Figure 1:**
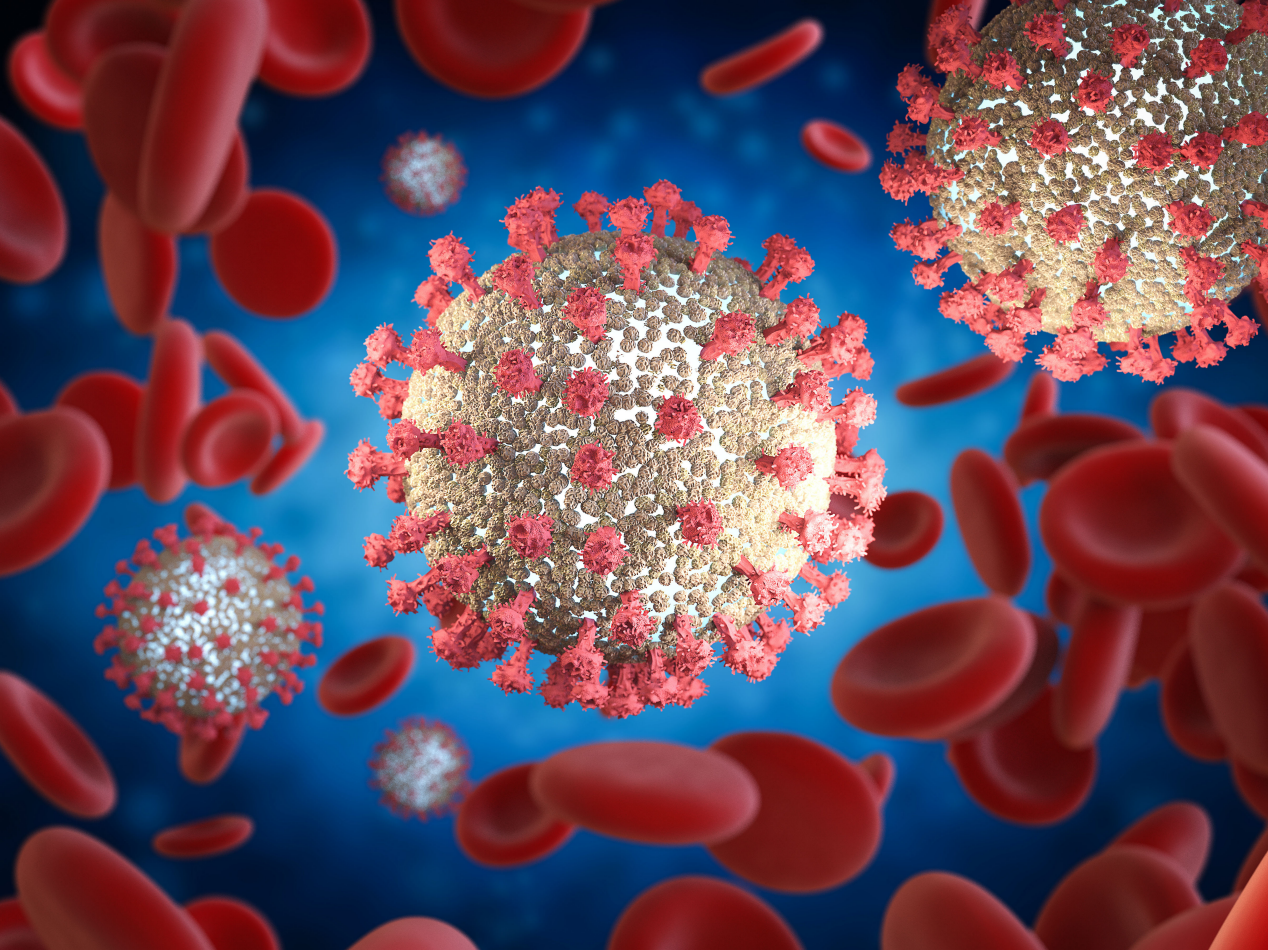
SARS-CoV-2. Copyright phonlamai (Kittipong Jirasukhanont)/Depositphotos Inc.

The SARS-CoV-2, which is the virus responsible for the disease, is one of the coronaviruses in the family of Coronaviridae. It belongs to genera Betacoronavirus and is the seventh coronavirus known to cause human diseases. The virus is a spherical or pleomorphic enveloped, non-segmented, single-stranded, positive-sense RNA virus (**Figure 1**). It has four main structural proteins: spike (S), membrane (M), envelope (E), and nucleocapsid (N) proteins (1, 7, 13).

Like other viruses, SARS-CoV-2 can mutate. The mutated virus is referred to as a variant of the original virus. Several variants of SARS-CoV-2 have been reported (e.g., Alpha, Beta, Delta, Epsilon, Eta, Gamma, Iota, Kappa, Lambda, Omicron, Theta, and Zeta). They were initially detected in countries like the United Kingdom, South Africa, Brazil, and India. Some variants are more contagious and aggressive and may show more resistance to the current vaccines. The Delta variant and then the Omicron variant created serious concerns in several countries, where they became the dominant variants affecting adults, adolescents, and children, responsible for spike in hospitalizations. Within a variant, there are also several sub-variants (e.g., BA.4 and BA.5 Omicron sub-variants).

The transmission of human-to-human mainly occurs from direct contact or by droplets spread by infected subjects through cough or sneeze. The survival of the virus in the environment ranges from a few hours to a few days, depending on the conditions. The nose, mouth, and ocular mucosa are the major ways of transmission.

The virus enters and infects the target cells by binding to cell membrane protein receptors. The most well-described cell membrane protein receptor is ACE2, a zinc metalloprotease (7, 9, 13, 14). ACE2 is expressed in multiple organs including pancreas and endothelium. It is abundant in the epithelia of the lung and intestine. After the binding of the virus spike protein to ACE2, there is an internalization of ACE2. The virus uses the genetic system of the host cell and replicates. At the end, the virus leaves the cell through exocytosis. The infected cells undergo apoptosis or necrosis, triggering inflammatory responses.

### Health consequences of COVID-19 infection

COVID-19 infection had influenced life at individual, familial, societal, and environmental levels through infection and confinement/isolation and had placed a significant burden upon healthcare worldwide (12). Different systems expressing ACE2 can be impacted by COVID-19 infection (e.g., respiratory, cardiovascular, neurological, gastrointestinal, and endocrine). COVID-19 infection causes alterations in the host immunological status including an increase in pro-inflammatory cytokines (e.g., interleukin-6 and tumor necrosis factor-α). The surge of pro-inflammatory factors (cytokine storm) can cause endothelial dysfunction and host organ damage (9, 15, 16).

Subjects infected with COVID-19 can be asymptomatic, have mild symptoms recovering within 1 to 2 weeks, or be severely affected with the ultimate risk of death. Common symptoms include fever, dry cough, dyspnea, arthralgia, myalgia, ageusia, and anosmia. COVID-19 symptoms can sometimes last several months. The damage to the lungs, heart, and brain increases the risk of long-term symptoms (long haulers). COVID-19 infection interferes with the onset and evolution of multiple endocrine and metabolic disorders (5–9, 17). For some endocrine/metabolic systems and diseases, information on pathophysiology and long-term outcome is relatively limited.

All age groups may be affected by COVID-19 infection. The disease is more severe in men (6, 18). Older subjects (> 65 years), black subjects, smokers, and subjects with immunodeficiency, cardiac and respiratory diseases, cancer, hypertension, diabetes, obesity, and dyslipidemia are considered high-risk populations (13, 19).

Social distancing and home confinement/isolation were the key public health recommendations during the COVID-19 pandemic. More than 4 billion people worldwide experienced the mobility restriction. The home confinement, self-isolation, and unemployment for a long period are responsible for alterations in lifestyle (e.g., unhealthy diet and reduced physical activity) and mental status (e.g., anxiety and depression). The confinement and isolation can impact access to health care (e.g., medications, physicians, and hospital beds) for the management of pre-existing medical conditions (e.g., heart disease, cancer, and diabetes) (12, 20). The changes in lifestyle can lead to insulin resistance and weight gain (overweight or obesity) and death may result from direct consequences of the viral infection, mental complications of confinement/isolation (risk of suicide), or aggravation of pre-existing diseases (4, 8, 20–22).

## Diabetes

### Prevalence of diabetes

Diabetes is a complex metabolic disease that results from deficiency of insulin secretion and/or action. It affects people regardless of country, age, or gender. Approximately half of the people living with diabetes are unaware of their condition. This proportion is much higher in low-income countries. Diabetes, both type 1 and type 2, is a major cause of morbidity and mortality in the world.

The prevalence of diabetes has risen significantly over the last several decades and is expected to rise dramatically in the years to come. In 2021, the global age-standardized prevalence of diabetes was 6.1% with 529 million people of all ages living with diabetes worldwide; type 2 diabetes accounted for more than 96% of the cases. In the USA, the numbers of subjects with type 1 and type 2 diabetes are around 2 and 35 million, respectively. It is expected that by 2050, the prevalence of diabetes will exceed 10% with more than 1.31 billion people living with diabetes. This increase will be mainly driven by type 2 diabetes which is primarily due to a rise in obesity (23).

### Health consequences of diabetes

**Figure 2:**
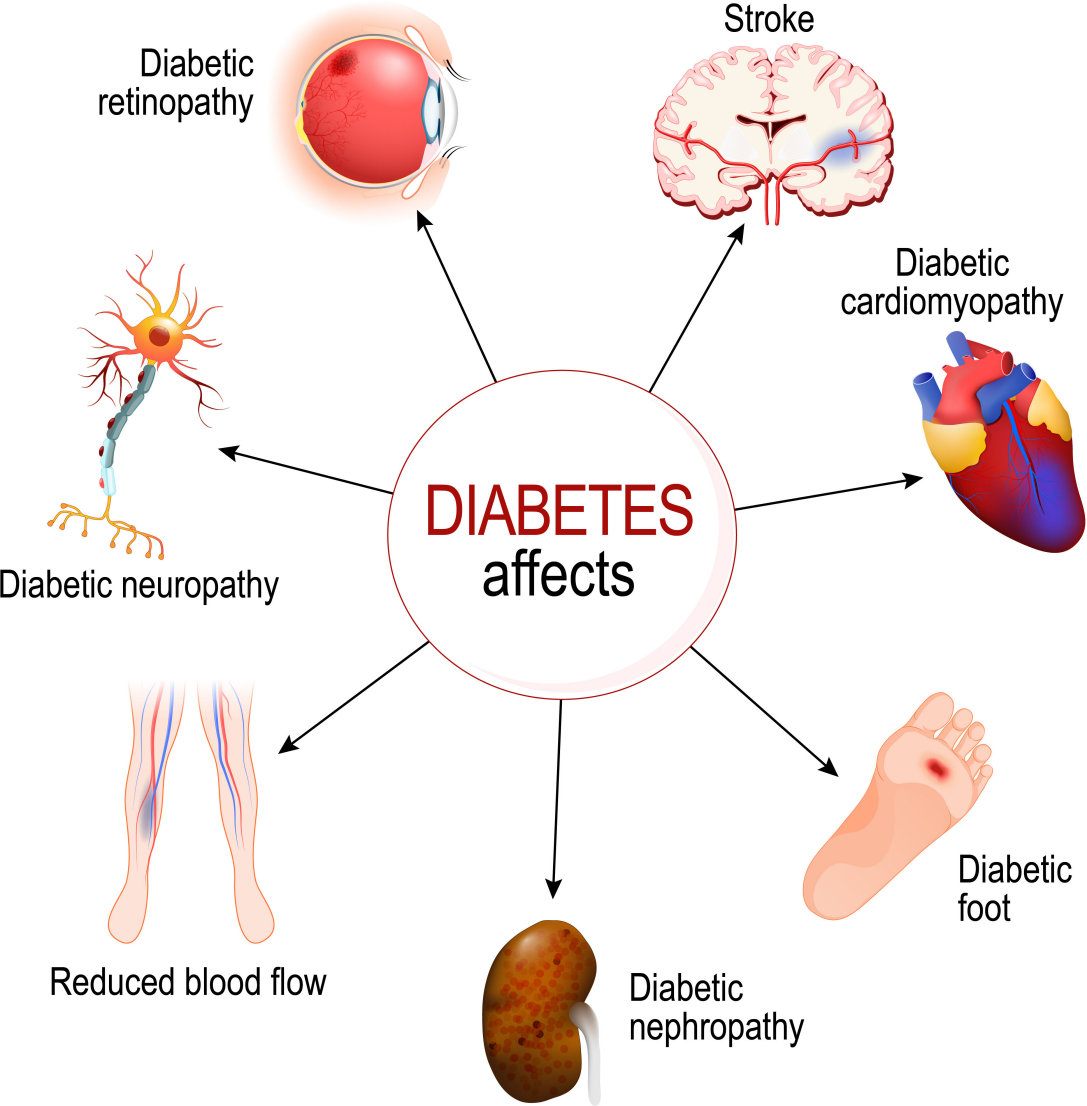
Diabetes negatively impacts several organs. Copyright edesignua (Tetiana Zhabska)/Depositphotos Inc.

Diabetes causes multiple complications including macrovascular (e.g., cardiomyopathy) and microvascular (e.g., neuropathy, retinopathy, and nephropathy) complications inflicting high cost on healthcare (**Figure 2**) (24).

Hyperglycemia associated with diabetes causes impaired immunity (e.g., reduced T lymphocytes response, reduced neutrophil function, and disorders of humoral immunity) (25). The frequency and severity of infectious diseases are higher in subjects with diabetes in comparison to those without diabetes. The risk of infection is enhanced in the elderly population (26).

## Impact of COVID-19 infection on diabetes

### Impact through metabolic changes due to confinement and isolation

COVID-19 infection can impact type 2 diabetes through alterations in adipose tissue mass, cytokines levels, and insulin sensitivity. The confinement and isolation of the COVID-19 pandemic promote unhealthy diet (e.g., overeating) and reduced physical activity, both leading to excess adiposity (overweight or obesity) especially in high-income populations, increased production of cytokines (systemic inflammation), and insulin resistance (8, 20, 21). These alterations can promote or aggravate type 2 diabetes.

Daily exercise (e.g., low to medium-intensity exercise) is essential for preventing the negative impact of inactivity and improving health (20, 22).

### Impact through cytokine storm

By causing a cytokine storm with the resulting release of multiple pro-inflammatory factors (e.g., interleukin-6 and tumor necrosis factor-α), insulin resistance, endothelial dysfunction, and damage of the pancreatic islets, COVID-19 infection contributes to the promotion or aggravation of type 1 or type 2 diabetes and complications of diabetes (16).

To reduce the risk of severe outcome of diabetes, it is crucial to achieve glycemic control. It is also important that subjects with diabetes be prioritized for COVID-19 vaccination.

### Impact through changes in healthcare services

COVID-19 infection has caused a major disruption in the management of subjects with diabetes. The services offered by healthcare systems had to be adapted accordingly. Routine in-person appointments have been minimized to avoid crowds in waiting rooms with the risk of infection. Outpatient management with remote advice and support services using phone calls, video calls, and e-mails have been recommended, promoted, and implemented (19, 27). Elective surgical procedures have been postponed when possible.

## Impact of diabetes on COVID-19 infection

### Impact through adipose tissue

Type 2 diabetes is closely related to overweight and obesity. Indeed, in subjects with type 2 diabetes, at least 85% have overweight or obesity, and among subjects with obesity, around 30% have type 2 diabetes. ACE2 is expressed in adipose tissue. With higher adiposity commonly associated with type 2 diabetes, more receptors (ACE2) are available for SARS-CoV-2, exposing subjects to COVID-19 infection. In addition, subjects with excess adiposity may experience a more serious COVID-19 infection through several mechanisms (e.g., inflammation, impaired immunity, mechanical lung dysfunction, impact of comorbidities, and vitamin D deficiency) (5, 7, 15, 16, 18, 19, 28–31).

Management of subjects with excess body weight and COVID-19 who require treatment in intensive care units can be challenging (e.g., difficulty for moving, for intubating, and for obtaining diagnostic imaging) (15). Subjects with overweight or obesity should have weight reduction using appropriate approaches and tools, when indicated (e.g., diet, exercise, behavioral change, drugs, medical devices, gut microbiome modulation, and bariatric surgery) (32–37).

### Impact through immune system

In subjects with uncontrolled diabetes, chronic hyperglycemia negatively impacts the immune system and increases the risk of COVID-19 infection and its morbidity/mortality (5–7, 13, 15, 18, 19, 27, 38).

Appropriate glycemic control is essential to reduce the risk of COVID-19 infection. The outpatient plasma glucose goal in case of COVID-19 infection is 72-144 mg/dL with a hemoglobin A1c goal less than 7%. Plasma glucose should be monitored at least twice daily.

### Impact through other complications of diabetes

The presence of other complications of diabetes such as endothelial dysfunction, cardiovascular disease, and nephropathy can be responsible for poor COVID-19 outcome (16).

### Impact through antidiabetic medications

Some antidiabetic medications are not suitable in severe cases of COVID-19 infection (16). Particularly, sodium-glucose cotransporter-2 inhibitors should be discontinued in subjects severely affected by COVID-19 infection and who are at risk of dehydration. In hospitalized subjects with severe COVID-19 infection, insulin is the preferred treatment for type 2 diabetes with the use of continuous glucose monitoring (6).

## Conclusion

There are bidirectional interactions between COVID-19 infection and diabetes. COVID-19 infection can impact the onset and/or the evolution of diabetes and diabetes can impact the onset and/or the evolution of COVID-19 infection.

COVID-19 infection has caused a major disruption in the management of subjects with diabetes. The medical services have been adjusted to the new situation. Routine in-person appointments can be reduced to avoid crowds in waiting rooms with the risk of infection. Outpatient care with remote advice and support services have been promoted. Because of the higher risk of mortality in subjects with diabetes who are infected by SARS-CoV-2, tight glycemic control and proper COVID-19 vaccination are essential in this population.

## Author contributions

HMH: Conceptualization, Writing – original draft.
